# Prevalence of Product Claims and Marketing Buzzwords Found on Health Food Snack Products Does Not Relate to Nutrient Profile

**DOI:** 10.3390/nu12051513

**Published:** 2020-05-22

**Authors:** Maddison Breen, Hollie James, Anna Rangan, Luke Gemming

**Affiliations:** Nutrition & Dietetics Department, Charles Perkins Centre, School of Life and Environmental Sciences, The University of Sydney, Sydney, NSW 2006, Australia; mbre6607@uni.sydney.edu.au (M.B.); holliejamesdietitian@gmail.com (H.J.); anna.rangan@sydney.edu.au (A.R.)

**Keywords:** health food, nutrient content claims, health claims, food labelling, nutrient profile, health star rating

## Abstract

Growth in the consumer health and wellness industry has led to an increase of packaged foods marketed as health food (HF) products. In consequence, a ‘health halo’ around packaged HF has arisen that influences consumers at point-of-purchase. This study compared product claims (nutrient content claims (NCC), health claims and marketing ‘buzzwords’) displayed on packaged HF snack products sold in HF stores and HF aisles in supermarkets to equivalent products sold in regular aisles (RA) of supermarkets. Product Health Star Rating (HSR), nutrient profile and price were also compared. Data were collected for 2361 products from three supermarket chains, two HF chains and one independent HF store in Sydney, Australia. Mann-Whitney U tests compared the product claims, HSR, nutrient composition and unit ($) price. HF snacks displayed significantly more product claims per product compared to RA foods (HSR ≤ 2.5), median (IQR) 5.0(4.0) versus 1.0(2) and (HSR > 2.5) 4.0(4.0) versus 3.0(4), respectively (*p* < 0.001). A significantly different HSR was evident between HF and RA snack products, median 2.5(0) versus 2.0(1.5), respectively (*p* < 0.001). HF snacks cost significantly more than RA snack foods, irrespective of product HSR (*p* < 0.001). These findings support the recommendation for revised labelling regulations and increased education regarding consumers food label interpretation.

## 1. Introduction

Since 2004, the sales of packaged foods in Australia have nearly doubled and are predicted to continue to climb at a steady rate [1]. Likewise, in the past decade, ready-to-eat snack foods have increased in popularity amongst the Australian population [2]. Packaged, ready-to-eat snack foods can be defined as foods that have undergone a degree of processing and are designed to be consumed in the original state purchased [3,4]. Most fall within the discretionary food category (junk food) characterised by their high energy, saturated fat, added sugar and sodium content [2,5,6,7]. Thus, daily consumption should be limited due to their link with overweight and obesity, cardiovascular disease, diabetes and other co-morbidities [2,5,8].

Contrary to the rise in non-communicable diseases, such as obesity [9], consumer awareness of diet related health consequences has advanced [10]. It is evident that consumers are making a deliberate effort to modify certain dietary behaviours with the aim of improving their overall health and wellbeing [11,12,13,14]. According to a Nielsen report, sales of packaged health food (HF) products increased by 82% in supermarkets from 2012–2014, and most HF consumers report they shop in specialty retail stores that stock HF products [15]. In response, food manufacturers are constantly developing new HF products to capitalise on consumer demand [1]. These products are predominantly sold in HF aisles of supermarkets and specialty HF stores, which typically market themselves as food retailers in the health and wellness sector. In both locations, HF products are marketed and labelled as being nutritionally beneficial and often natural, organic or environmentally sustainable. Between 2012–2014, the sales of products with ‘natural’ or ‘organic’ claims grew by 24% and 28%, respectively [15]. Correspondingly, the value of ‘natural’ products has been influenced by consumer choice, with natural non-sugar sweetened product sales increasing by 186%, due to perceived health benefits, while artificially sweetened product sales decreased by 12% [15].

While the term healthy is defined as “beneficial to one’s physical, mental, or emotional state: conducive to or associated with good health or reduced risk of disease” [16], the measured healthfulness of a product is difficult, given the differing attributes associated with health by consumers. Research indicates that consumers choose products that advertise ‘healthy’ qualities to attempt a more nutritionally balanced lifestyle [13]. Research also shows that consumers believe organic, gluten free and/or more expensive products to be healthier than the alternative [11,12,13,17,18]. Thus, a ‘health halo’ can exist [19,20], where consumers assume foods that are perceived to be ‘healthy’ have greater health benefits, more nutrients and fewer health risks than may actually be true [21,22,23,24].

With the aim to assist consumers in interpreting food labels more appropriately, the Health Star Rating (HSR) was implemented in Australia as a voluntary front of pack labelling (FoPL) scheme in 2014 [25]. Consumers prefer FoPL, including HSR and nutrition content claims (NCCs) over nutrition information panels (NIPs), because of their simplicity [24,26,27]. NCCs and health claims are images or words on product packaging that highlight particular properties and/or their health impact. Although the FoPL labels are strictly controlled by Food Standards Australia New Zealand (FSANZ), they may also contribute to the health halo effect when placed on products with other marketing messages with little to no restrictions, otherwise known as ‘buzzwords’, or when placed on products that are not necessarily healthier [21,23,26,28,29,30,31,32]. As such, questions have been raised regarding the use of NCCs and the HSR on packaged foods [33,34,35,36,37]. 

Due to the consumer confusion and vagueness of the term ‘health’ or ‘healthy’, products in the UK are not permitted to be labelled as such [38], while FSANZ does not mention this term specifically in nutrient and health claim regulations, neither permitting nor preventing its use [28]. Thus, a climate exists in food labelling where people who want to make healthier choices, by seeking healthy food products, may find it difficult to appropriately determine their value [39,40,41,42]. Concurrently, there has been significant growth in HF snack products sold in supermarket HF aisles and specialty HF stores. However, as there are no governing criteria of what can be stocked in these locations, the true health benefits of these products are largely unknown.

Accordingly, the primary aim of this study was to examine and compare the use of NCC, health claims and marketing ‘buzzwords’ on packaged HF snack products sold in supermarkets and specialty HF stores to equivalent products sold in RA of supermarkets. A secondary aim was to compare the nutrition profile and cost of these products.

## 2. Materials and Methods 

### 2.1. Data Collection

Ethics approval was not required for the completion of this study. Data were collected from March 2018 to August 2019 as part of an audit of commercially available packaged snack foods in Australia [43]. Data were collected from the four major Australian supermarkets in the Sydney metropolitan area: Woolworths, Coles, Aldi and IGA. To capture additional HF snack products not sold in the major supermarkets, data were also collected from two national HF store chains, Go Vita and Healthy Life. To ensure data collection had reached saturation of the market, one large independent HF store was chosen at convenience for data collection. Several other HF stores across Sydney were subsequently visited to verify completeness of data collection. Assessment of the HF store’s products indicated saturation was reached and therefore not used in the study. Not all supermarkets had entire dedicated HF aisles. Thus, HF aisle was defined as the aisle (complete or partly) that contained ‘health foods’, ‘gluten free products’ or ‘sports nutrition products’ as per aisle signage. Products located in all other aisles of supermarkets that contained a gluten free label were classified as RA foods. 

Researchers captured images of the front and back packaging, ingredient list, Nutrition Information Panel (NIP) and barcode of all products using smartphones in-store. The products were classified into seven main categories and thirteen sub-categories (detailed in supplementary material Table S1). Once the data for HF snack foods was recorded, equivalent or ‘like’ products were sourced from the regular aisles (RA) of the supermarkets. 

Product claims were divided into three categories; nutrient content claims (NCC), health claims and ‘buzzwords’. Nutrient content and health claims were defined using the FSANZ definitions [28]. All other claims were categorised as ‘buzzwords’ (claim descriptions in supplementary material Table S2). Nutrition information from the NIP and full unit price values ($AUD) of all products were recorded and standardised per 100 g. Where the same products were available across multiple supermarkets or stores, price was taken from Coles or Woolworths, the first location where the product was recorded. All data were manually entered into an online database. Ready-to-eat packaged snack foods that were still wholefoods and/or were only minimally processed were excluded from collection, e.g., dried fruit and nut snack packs.

Data cleaning was carried out and duplicates of the same product within the same store type and duplicate products with different package sizes were removed from the database. The smallest package size was kept in the database and the unit cost was calculated from this. All outliers were checked against the original images. Any NIP values that stated nutrient content as <X, values were input as X - 1 for analysis, e.g., < 10 g was input as 9 g. 

For those products that did not specify an HSR, the HSR was calculated using the Australian Government’s HSR calculator (HSRC) [44]. Negative nutrients include energy (kJ), saturated fat, sugar and sodium, which accrue points, while positive nutrients such as protein, fibre and fruit, vegetable, nut and legume (FVNL) content deduct points; the higher the product score, the lower the HSR. Although, as many products did not declare ingredient percentage of product weight, the FVNL scores were estimated using a previously tested method [8]. As per other systematic analyses of the Australian food supply [37], for those products that did not specify fibre content in the NIP, a value was estimated from the nearest matched product from the AUSNUT 2011–2013 Food Nutrient database [45]. A sensitivity analysis was performed to determine whether these methods affected the derived fibre values and the derived HSR outcome.

### 2.2. Data Analysis

Data analysis was conducted using IBM SPSS Statistics Version 24 (2016), Armonk, NY, USA. The proportion (%) of products displaying NNC claims, health claims and buzzwords on HF snack products and equivalent RA foods was calculated and presented using descriptive statistics. The HSR was used to broadly classify product ‘healthfulness’; products were classified as having an HSR ≤ 2.5 or >2.5 for NCC, health claim and buzzword comparisons. The data were checked for normality and found to be non-normal distribution; therefore, the median and interquartile range were used. The product claims, nutrient composition and unit price ($) per 100 g were compared between HF snack products and equivalent RA foods using Mann-Whitney U tests. A *p*-value of <0.001 was considered statistically significant due to the large number of tests undertaken. The HSR was used to group products for unit price comparisons using descriptive statistics. 

## 3. Results

A total of 2361 snack products were collected; 1251 sold in RA and 1110 HF products sold in “health food” aisles of supermarkets and specialty “health food” stores; 621 products from HF aisles and 489 products from HF stores. The HSR was derived for 80% of RA products and 82% HF products. The fibre content was derived for 53% of RA and 11% of HF snack products. The sensitivity analysis revealed no apparent differences between original and derived fibre and HSR values; therefore, derived HSR values were used in the analysis. The largest category for HF snacks was snack bars (35%) and for RA products was confectionary (19%).

### 3.1. Nutrient Content Claims, Health Claims and ‘Buzzwords’

A total of 8155 product claims were recorded, 5626 for HF snack products (2726 from HF aisles and 2900 from HF stores) and 2529 for RA snack products. Overall, 94% of the HF snack products and 73% of RA snack products reported/displayed NCC, health claims or ‘buzzwords’. 

Table 1 shows the proportion that different NCC, health claims or ‘buzzwords’, directly or indirectly related to health, were displayed on HF and RA snacks products (number of NCC, health claims and buzzwords/total HF or RA snack products). 

‘Gluten free’ was the most common NCC displayed on both HF and RA snack products. ‘Vegan’ was the most common buzzword used on HF snack products and ‘no artificial’ was the most common for RA snack products. Other buzzwords, including ‘no artificial’, ‘natural’, ‘dairy free’, ‘organic’ and ‘allergen free’, were also frequently displayed (>25%) on HF snack products. ‘Dairy free’ was the only other buzzword displayed frequently (>25%) on RA snack products. Due to small individual numbers, a wide range of “other claims” were grouped together. At least one of these “other claims” were present on 100% of HF snack products and 39.6% of RA snack products. 

Table 2 compares the proportion of HF and RA snack products with an HSR ≤ 2.5 or > 2.5, with 50% of HF and 25% RA snack products scoring an HSR > 2.5. Overall (all categories), HF snack products displayed significantly more NCC, health claims and buzzwords per product compared to RA products, median 5.0 versus 1.0 (HSR ≤ 2.5) and 4.0 versus 3.0 (HSR > 2.5), respectively (*p* < 0.001). For those products with an HSR ≤ 2.5, HF snacks displayed significantly more product claims per product for all categories. Similarly, for those products with an HSR > 2.5, HF snacks displayed significantly more product claims per product for all categories excluding chips and sweet biscuits was significantly lower. Small sample sizes (*n* < 10) for these two categories, chocolate and confectionary, were evident.

### 3.2. Nutrient Composition and HSR

Table 3 shows the median HSR and nutrient composition of HF and RA snack products. Overall (‘all categories’), the median HSR for HF snack products was significantly higher than RA products, 2.5 versus 2.0, respectively (*p* < 0.001). Compared to RA snacks, the median HSR for HF snack products was significantly higher for all categories, except beverages and confectionary. Overall (‘all categories’), HF snack products were significantly higher in protein, total fat and fibre and RA snack products were significantly higher in carbohydrates and sugar (*p* < 0.001). No difference in energy, saturated fat or sodium was evident. For HF snack products, all categories, except beverages and confectionary, were significantly higher in fibre than RA products (*p* < 0.001). 

### 3.3. Price

**Figure 1:**
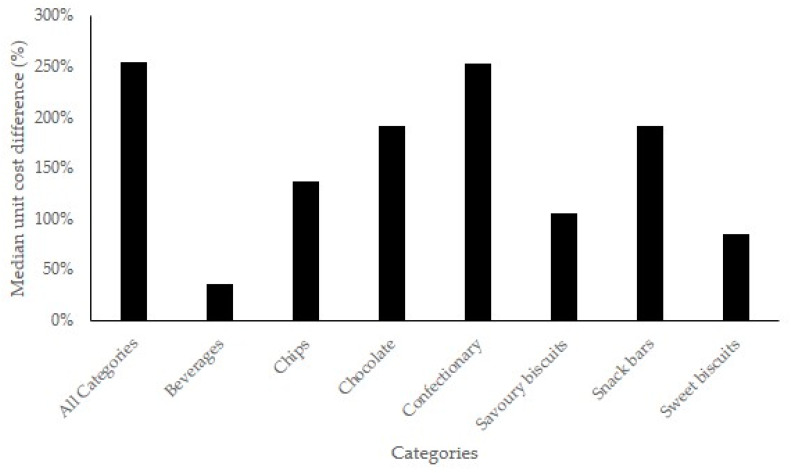
Median unit cost ($) per 100 g percent difference (%), per product category, between health food (HF) snack products sold in supermarkets and specialty HF stores compared to equivalent products sold in regular aisles (RA) of supermarkets.

Figure 1 shows HF snack products in all food categories were substantially more expensive than RA foods. The largest overall price difference was between HF snack products and RA products was confectionary (253%). Median unit price ($) of HF snack products was significantly higher than RA products for all product categories (supplementary material Table S3).

**Figure 2:**
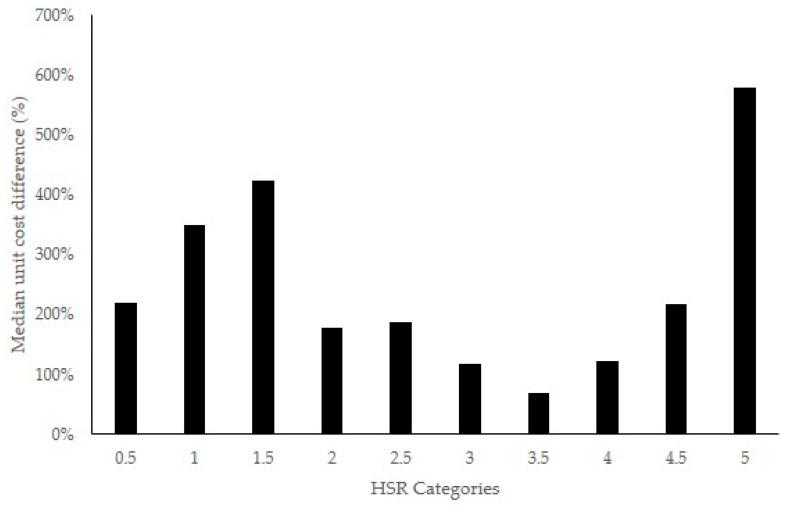
Median unit cost per 100 g percent difference (%), per HSR category, between health food (HF) snack products sold in supermarkets and specialty HF stores compared to equivalent products sold in regular aisles (RA) of supermarkets.

Figure 2 shows unit cost ($) differences between product types, per HSR category. Health foods were substantially more expensive for all HSR categories. No clear trend between unit price ($) and HSR was evident. The greatest price difference was between HF and RA snack products scoring highest HSR of 5.0 (579%), followed by products scoring the lowest three HSR of 1.5, 1.0 and 0.5.

## 4. Discussion

This study sought to examine and compare NCC, health claims and ‘buzzwords’ displayed on pre-packaged snack HF products sold in supermarkets and specialty health food stores to equivalent products sold in RA of supermarkets. Secondary aims were to compare the nutrition profile and cost. The main findings of this study revealed manufactures of HF snack products use significantly more NCC, health claims and buzzwords to market their products compared to equivalent products sold in RA of supermarkets irrespective of their overall ‘healthfulness’. Surprisingly, the greatest use of NCC, health claims and buzzwords was found on HF snack products with HSR ≤2.5 (median five claims per product). In contrast, equivalent products sold in RA only displayed one NCC, health claim or buzzword per product, revealing the presence and quantity of claims often does not relate to product healthfulness, and particularly for HF snack products, may instead encourage consumption of foods associated with increased health risks misleading consumers [8,21,23,26,33,46,47]. Furthermore, it must also be noted that the health halos that NCC may help create, also applies to the absence of misunderstood constituents, such as gluten. In the current study, a substantially higher proportion of HF snack products were labelled gluten free, despite 50% of products displaying an HSR ≤ 2.5. This is not surprising considering consumers often consider gluten free foods to be more beneficial to health [11,48].

The HSRC algorithm was used as a proxy to estimate a foods ‘healthfulness’ [44]. Overall, HF snack products were marginally superior to equivalent products sold in RA with a small but significant difference evident, median 2.5 versus 2.0, respectively. The slightly higher HSR achieved by HF snack products is likely attributed to greater fibre and lower sugar found across several categories, but no differences were evident for energy, saturated fat or sodium, all noted to be of concern by the World Health Organisation as detrimental to human health [9]. The significantly higher total fat and lower carbohydrate content in HF snack products was also notable and likely attributed to increased use of plant-based fats, nuts and seeds evident from the product ingredient lists. While our research used the mid-point of HSR system 2.5/5 to broadly classify foods into two distinct groups when examining differences in product labelling, a higher HSR cut-off ≥ 3.5 has been used by others to more clearly distinguish ‘healthier’ food choices and reduce the likelihood of discretionary foods being classified as healthy foods [7,37,49]. Accordingly, neither HF snacks nor equivalent RA products would meet this cut-off. This may be expected for products in RA, which are not always manufactured or viewed as the healthier options, but emphasises the concern surrounding HF snack products. Thus, the median HF score of 2.5/5 should be considered a minimum passing grade at best, a marked difference from the health halo surrounding products marketed as health foods [34].

Despite the limited research in this area, overall, these results were consistent with previous findings. Studies that have examined a range of products with and without NCC and health claims, or claimed to be ‘organic’, found that most products showed no difference in overall nutrient profile [8,21,23,26,33,46,47,50]. Pertinent to our own findings, Hughes et al. [33] found that a large number of NCC and health claims used on Australian products did not meet FSANZ nutrient profiling criteria [51] and this is likely true for a proportion of products examined in this study.

Previous literature has sought to determine appropriate FoPL to improve consumer perception of a products nutrition, without the strong influence of NCC, health claims and buzzwords, though not specifically for HF products [21,52,53,54,55,56,57,58,59]. Research shows that consumers have a poor understanding of food labels and cannot appropriately interpret the NIP [39,40], especially when a product claim is present [41,42]. In addition, qualitative literature has consistently found that consumers believe labels such as ‘organic’ [12,17,60], ‘natural’ and ‘not artificial’ [13] indicate that products are more nutritious [17] and are lower in sugar, fat and sodium [13]. Likewise, the placement of snack products within HF aisles and specialty HF stores marketed as ‘health foods’ (for which there is limited regulation) may act as a buzzword itself influencing consumer purchases. Evidence suggests the “reductive style” [55] of the HSR reduces consumer inclination to buy unhealthy products and guide more accurate interpretations [21,47,59,61]. However, our data show most products did not display HSRs. Due to the voluntary nature of HSR, manufacturers may preferentially display HSRs on healthier products, therefore increasing consumer reliance on other product labels and claims [62]. Additionally, in agreement with other research, our data show that discretionary foods can obtain HSR scores >2.5, potentially distracting from the consumption of foods from the five food groups and showing poor alignment with the Australian Dietary Guidelines [34,35,36,37,49]. Together, these findings provide strong evidence for revised labelling regulations, and increased education initiatives for consumers on how to interpret nutrition labels, to make informed purchasing decisions [14,54].

Though the number of NCC, health claims and buzzwords that HF snack products displayed did not correlate to a higher HSR, they may partly explain the higher price of HF products. The majority of HF snack products in some categories claimed to be vegan, organic, environmentally conscious or made ‘good sugar’ claims. Thus, in conjunction with (likely) smaller production, the cost of organic and alternative ingredients such as coconut oil, increased use of nuts, wheat and cane sugar alternatives are likely more expensive. However, the large price differences observed are not likely due to production costs alone. In addition, the price premium for purchasing HF products was not related to the HSR. Other research has reported similar findings, with foods labelled ‘organic’ found to have a similar nutrition profile but cost significantly more than those that are not [50,63,64]. This is of significance as the use of ‘buzzwords’, along with higher price points, have been found to strongly influence consumers and generate a misleading health halo effect [13,15,17,29,31,32,65]. Consumers should have the right to seek and pay a premium for ethical, organic and sustainable food options, though this should not be confused with purchasing healthier choices.

When interpreting these data, limitations must be considered. The study was limited to snack foods, and therefore did not assess all products found in HF stores and aisles, such as cereal products. However, the remaining products (excluding whole foods e.g., nuts) do represent most other products found in HF stores and HF aisles. Over 80% of the HSR for all products from all store types were derived using the HSRC [44]. Furthermore, some fibre values also had to be derived for HSR calculation. Despite the sensitivity analysis conducted, and a validated approach used by others [8,62], the derived values are only estimates and might differ from the true values. Additionally, several researchers have raised concerns regarding the HSRC to appropriately assess foods ‘healthfulness’ [34,35,36,37]; thus, the system is not without limitations. ‘Buzzwords’ regarding environmentally conscious claims were grouped within the overall results for interpretation but do not directly imply a product is healthier. Some values may also be skewed due to the placement of supermarket products. For example, gluten free sections are often contained within HF aisles; thus, our HF data contains both formulated gluten free product alternatives such as gluten free biscuits and other HF products simply marketed as gluten free along with other buzzwords. Thus, the marketing intent of the gluten free label may be different between products. Due to nutrition labelling regulations in Australia, added sugars were not distinguished from natural sugars. Future research could analyse the difference between added and natural sugars between these store types using other datasets. Finally, these data are a snapshot of products from the Sydney metropolitan area, across a certain time. Due to constant fluctuation in product availability and pricing, the packaged food supply may have changed at time of publication. However, with over 2000 products analysed, the study has provided a reliable sample, and thus comparison, of packaged snack foods in HF stores, HF aisles and regular aisles in 2019.

## 5. Conclusions

The main findings of this study revealed manufactures of HF snack products use substantially more NCC, health claims and ‘buzzwords’ to market their products compared to equivalent products sold in RA of supermarkets irrespective of their overall ‘healthfulness’, and may actually encourage the consumption of foods associated with increased health risks, misleading consumers. Although the nutrition profiles of HF snack products were marginally better than equivalent products found in RA, overall, the HF snack products examined in the study often received low HSR ≤ 2.5, with most being discretionary choices, a marked difference from the consumer perception and health halo surrounding HF products. Health food snack products were also found to be substantially more expensive, but this was not consistent with the ‘healthfulness’ of a product. If consumers pay a premium for ethical, organic and sustainable foods, they should not be confused with purchasing foods that are healthier. Thus, the findings of this research provide strong evidence to support recommendations for revised labelling regulations, particularly surrounding HF snack products. Increased efforts to educate consumers on label reading are required to help consumers make informed and healthy choices.

## Figures and Tables

**Table 1:** The proportion (%) of snack products that display nutrient content claims, health claims or ‘buzzwords’ on health food (HF) snack products sold in supermarkets and specialty HF stores, and equivalent products sold in regular aisles (RA) of supermarkets. FSANZ: Food Standards Australia New Zealand.

	**Health Foods (%)**	**Regular Aisle Foods (%)**
**Nutrient content claims**		
Gluten free	66.8	12.5
Sugar (e.g., no added sugar, low sugar etc.)	24.2	7.6
Fibre (e.g., source of fibre)	16.5	6.3
Protein (e.g., source of protein)	10.2	4.1
Fat (e.g., low fat, fat free etc.)	5.2	2.8
Sodium (e.g., low sodium/salt, salt reduced etc.)	2.7	0.5
**Health claims**		
All “health claims” (as per FSANZ)	2.5	2.4
**Buzzwords**		
“No Artificial” (e.g., no artificial colours, Flavours and/or preservatives)	27.6	34.5
Vegan	36.6	1.8
Natural	30.1	6.6
Organic	26.9	2.7
Dairy Free	27.0	28.9
Non-GMO	17.8	1.4
Wholegrain (e.g., Source of wholegrain)	5.0	6.6
Allergen free	26.6	1.4
Raw	7.8	0.1
Paleo	3.7	-
Keto	1.3	-
Environmental (e.g., green energy, Sustainable)	15.6	3.9
Superfood (e.g., ‘supergrain’, antioxidant, activated)	8.1	0.9
Nutritious (e.g., healthy, wholefood)	10.5	2.9
Good fats (e.g., good natural fats, omega 3)	5.3	4.6
Good sugars (e.g., natural sugars, fructose free, no refined sugar)	6.3	3.0
Made in Australia	4.2	29.7
All “other claims” directly and indirectly related to health and wellbeing (e.g., boost your inner health, burn fat, clean, FODMAP * friendly, low GI, made from real fruit, minimally processed, supports immune function, tone body…)	100	35.6

* Fermentable Oligosaccharides, Disaccharides, Monosaccharides and Polyols.

**Table 2:** Comparison of the median (IQR) product claims displayed on health food (HF) snack products sold in supermarkets and specialty HF stores to equivalent products sold in regular aisles (RA) of supermarkets, by product category for products with an HSR ≤ 2.5 and products with an HSR >2.5. Differences in median (IQR) claims displayed were assessed via Mann-Whitney U tests.

	Median Number of Product Claims Per Product with HSR ≤ 2.5			Median Number of Product Claims Per Product with HSR > 2.5		
	(N)	RA	HF	(N)	RA	HF
**All categories**	RA (*n* = 933) HF (*n* = 555)	1.0 (2.0)	5.0 (4.0) *	RA (*n* = 318) HF (*n* = 555)	3.0 (4.0)	4.0 (4.0) *
**Beverages**	RA (*n* = 54) HF (*n* = 49)	2.5 (3.0)	4.0 (4.0) *	RA (*n* = 89) HF (*n* = 46)	4.0 (3.5)	4.0 (2.0) *
**Chips**	RA (*n* = 97) HF (*n* = 61)	3.0 (2.0)	4.0 (5.0) *	RA (*n* = 11) HF (*n* = 133)	3.0 (2.0)	5.0 (2.0)
**Chocolate**	RA (*n* = 222) HF (*n* = 176)	1.0 (1.0)	6.0 (4.0) *	RA (*n* = 0) HF (*n* = 5)	-	4.0 (3.0)
**Confectionary**	RA (*n* = 193) HF (*n* = 34)	1.0 (1.0)	4.0 (4.0) *	RA (*n* = 49) HF (*n* = 6)	1.0 (1.0)	2.0 (2.5) *
**Savoury biscuits**	RA (*n*=108) HF (*n*=37)	2.0 (3.0)	2.0 (3.5) *	RA (*n* = 59) HF (*n* = 82)	3.0 (2.0)	6.0 (5.0) *
**Snack bars**	RA (*n* = 70) HF (*n* = 114)	3.0 (3.0)	5.0 (4.0) *	RA (*n* = 104) HF (*n* = 271)	3.0 (3.0)	4.0 (4.0) *
**Sweet biscuits**	RA (*n* = 189) HF (*n* = 84)	1.0 (2.0)	5.0 (3.5) *	RA (*n* = 6) HF (*n* = 12)	6.0 (4.0)	1.5 (2.5) *

Product claims include all nutrient content claims, health claims and ‘buzzwords’. Health Star Rating abbreviated to HSR. Regular aisles abbreviated to RA. Health foods Abbreviated to HF. * Denotes *p*-value < 0.001.

**Table 3:** Comparative analysis of the differences in median (IQR) for HSR and nutrient content between health food (HF) snack products sold in supermarkets and specialty HF stores compared to equivalent products sold in regular aisles (RA) of supermarkets. Differences in median (IQR) nutrient values were assessed via Mann-Whitney U tests.

	HSR	Energy (kJ/100 g)	Protein (g/100 g)	Total Fat (g/100 g)	Saturated Fat (g/100 g)	CHO (g/100 g)	Sugar (g/100 g)	Sodium (mg/100 g)	Fibre (g/100 g)
**All categories**									
RA (*n* = 1251)	2.0(0.0)	1850(560)	5.6(4.3)	14.9(24.2)	4.3(11.7)	62.1(26.3)	25.9(41.5)	105(300)	2.3(2.8)
HF (*n* = 1110)	2.5(1.5) *	1805(553)	8.0(9.8) *	18.9(20.6) *	4.3(11.7)	44.8(41.5) *	11.1(27.4) *	153(327)	7.2(7.1) *
**Beverages**									
RA (*n* = 143)	3.5(2.5)	181(242)	0.9(2.6)	0.9(1.7)	0.9(1.0)	7.8(7.6)	6.3(7.1)	10(40)	0.2(0.9)
HF (*n* = 95)	2.5(3.0)	112(165)	0.7(3.9)	0.2(1.1)	0.2(0.9)	2.4(4.8) *	2.1(5.0) *	5.0(58.6)	0.4(0.9)
**Chips**									
RA (*n* = 108)	2.0(1.5)	2100(160)	6.7(1.8)	27.8(8.7)	4.3(10.5)	56.5(8.5)	2.3(2.4)	576(249)	3.5(1.7)
HF (*n* = 194)	3.5(2.0) *	1960(300) *	8.6(11.1) *	21.3(11.8) *	2.1(1.9) *	57.1(17.7)	2.8(5.9)	465(382) *	7.2(6.8) *
**Chocolate**									
RA (*n* = 222)	0.5(0.0)	2200(253)	6.0(2.3)	29.8(11.1)	17.6(6.1)	57.8(14.3)	50.9(13.2)	69(52)	2.3(1.1)
HF (*n* = 181)	1.0(1.5) *	2320(410) *	7.0(3.1) *	41.9(11.3) *	24.0(10.2) *	37.6(19.7) *	26.7(20.4) *	50(75) *	9.3(7.5) *
**Confectionary**									
RA (*n* = 242)	1.5(1.5)	1460(253)	1.0(2.7)	1.0(1.1)	1.0(0.9)	81.4(17.8)	51.2(33.3)	23(59)	0.0(1.0)
HF (*n* = 40)	2.0(1.0)	1462(224)	0.35(4.4)	0.1(0.9) *	0.0(0.9) *	80.5(15.7)	49.4(28.2)	61(65)	0.0(1.9)
**Savoury biscuits**									
RA (*n* = 167)	2.5(1.0)	1790(250)	8.9(3.2)	10.5(11.5)	2.0(3.3)	70.1(12.9)	2.7(3.7)	628(360)	3.6(1.1)
HF (*n* = 119)	3.0(1.5) *	1750(347)	9.0(5.5)	9.9(16.8)	1.6(3.9)	67.9(28.4)	1.8(3.5)	570(345)	4.4(9.1) *
**Snack bars**									
RA (*n* = 174)	3.0(1.5)	1795(370)	9.5(9.1)	17.2(17.5)	5.5(4.2)	51.5(25.0)	23.6(11.7)	126(186)	7.0(3.6)
HF (*n* = 385)	3.5(2.0) *	1670(422) *	15.0(21.7) *	16.2(14.5)	4.8(5.0)	37.3(40.6) *	19.8(27.4)	110(232)	9.1(4.9) *
**Sweet biscuits**									
RA (*n* = 195)	1.0(1.0)	2040(240)	5.4(1.8)	21.4(9.7)	11.9(7.2)	66.6(6.2)	34.6(12.6)	240(187)	1.9(1.2)
HF (*n* = 96)	1.5(1.0) *	1920(288 ) *	5.0(2.4)	22.3(8.7)	11.3(8.1)	62.1(13.8) *	26.1(14.9) *	221(220)	3.9(3.6) *

* Denotes *p*-value < 0.001. Regular aisles abbreviated to RA. Health foods abbreviated to HF.

## Supplementary Materials

The following are available online at https://www.mdpi.com/2072-6643/12/5/1513/s1, Table S1. Product category and subcategory descriptions. Table S2. Product claim categories. Table S3. Comparative analysis of median unit cost ($/100g) between health food (HF) snack products sold in supermarkets and specialty HF stores compared to equivalent products sold in regular aisles (RA) of supermarkets.
