# Supplementary material for: Prevalence of Product Claims and Marketing Buzzwords Found on Health Food Snack Products Does Not Relate to Nutrient Profile
Source: Nutrients. 2020 May 22;12(5):1513. doi: 10.3390/nu12051513 (PMC7284863; doi:10.3390/nu12051513)
Supplement: Supplementary file 1 [file nutrients-12-01513-s001.pdf]

Table S1. Product category and subcategory descriptions.

Product category	Category description
**Beverages, all**	
Beverages, fermented	Kombucha, kefir and other pro/pre-biotic beverages
Beverages, fruit-based	Juice and other beverages where fruit is the main ingredient
Beverages, milk-based	Smoothies, flavoured milk, iced coffee and other beverages where milk is the main ingredient
Beverages, protein	Specifically declared high protein beverages and other diet/sports beverages
**Chips, all**	
Chips, extruded	All chips made from cereal flours, legume flours, vegetable flours or starches
Chips, grain-based	Corn chips, chips where the main ingredient is wheat, rice or oats and popcorn
Chips, potato	All vegetable chips, including potato, sweet potato, beetroot and broccoli, all legume chips, including chickpea and fava bean and fruit chips
**Chocolate, all**	All chocolates, including chocolate coated nuts and dried fruit
**Confectionary, all**	Lollies, licorice, toffees, sugar coated nuts and dried fruit
**Savoury biscuits, all**	Plain and flavoured rice crackers and crispbreads
**Snack bars, all**	
Snack bars, cereal-based	Snack bars where cereals are the main ingredient e.g., oats
Snack bars, fruit-based	Snack bars where fruit is the main ingredient e.g., dates
Snack bars, nut-based	Snack bars where nuts or seeds are the main ingredient
Snack bars, protein-based	Snack bars specifically declared as high protein, or sold as a sports nutrition or diet bar
**Sweet biscuits, all**	
Sweet biscuits, chocolate	All chocolate biscuits and chocolate coated biscuits
Sweet biscuits, other	All other types of biscuits including plain shortbread and cream filled

Table S2. Product claim categories.

Product claim category	Category description
**Nutrient content claim***	Any claim indicating the presence or absence of a nutrient or bioactive substance, for example 'no added sugar', 'low sodium', '97% fat free' etc.
**Health claim***	Any claim that claims a relationship between a nutrient and an effect on health or between a nutrient and a disease, for example 'fibre for a healthy digestive system'
**'Buzzword'**	Any other claim that does not refer to a nutrient or effect on health, for example 'organic', 'raw', 'natural', 'vegan' etc.

*According to the FSANZ definitions.

Table S3. Comparative analysis of median unit cost ($/100g) between health food (HF) snack products sold in supermarkets and specialty HF stores compared to equivalent products sold in regular aisles (RA) of supermarkets.

		**RA**		**HF**	**p-value**
	**N**	**Median (IQR) ($/100g)**	**N**	**Median (IQR) ($/100g)**	
**All categories**	1251	1.68(1.66)	1110	5.94(5.14)	<0.001*
**Beverages**	143	0.89(0.45)	95	1.21(0.28)	<0.001*
**Chips**	108	1.89(1.84)	194	4.47(3.68)	<0.001*
**Chocolate**	222	3.02(1.75)	181	8.80(5.45)	<0.001*
**Confectionary**	242	1.73(4.13)	40	6.11(4.80)	<0.001*
**Savoury biscuits**	167	1.95(1.7)	119	4.00(2.30)	<0.001*
**Snack bars**	174	2.4(0.88)	385	7.00(3.33)	<0.001*
**Sweet biscuits**	195	1.58(1.11)	96	2.92(2.69)	<0.001*

*Denotes p-value <0.001. Regular foods abbreviated to RF. Health foods abbreviated to HF.
